# A *p-tert*-Butyldihomooxacalix[4]arene Based Soft Gel for Sustained Drug Release in Water

**DOI:** 10.3389/fchem.2020.00033

**Published:** 2020-02-28

**Authors:** Hao Guo, Runmiao Zhang, Ying Han, Jin Wang, Chaoguo Yan

**Affiliations:** ^1^School of Chemistry and Chemical Engineer, Yangzhou University, Yangzhou, China; ^2^School of Chemistry and Chemical Engineer, Nantong University, Nantong, China

**Keywords:** calixarenes, gel, controlled release, macrocyclic compounds, Ugi reaction

## Abstract

*P*-*tert*-butyldihomooxacalix[4]arene is a well-known calix[4]arene analog in which one CH_2_ bridge is replaced by one -CH_2_OCH_2_- group. Thus, dihomooxacalix[4]arene has a slightly larger cavity than that of calix[4]arene and usually possesses a more flexible cone conformation, and the bridged oxygen atom might provide additional binding sites. Here, we synthesized a new functional *p*-*tert*-butyldihomooxacalix[4]arene **1** through Ugi reaction with good yield (70%), starting from condensed *p*-*tert*-butyldihomooxacalix[4]arene *O*-alkoxy–substituted benzaldehydes, benzoic acid, benzylamine, and cyclohexyl isocyanide. Proton nuclear magnetic resonance spectroscopy (^1^H NMR), ^13^C NMR, IR, and diffusion-ordered ^1^H NMR spectroscopy (DOSY) methods were used to characterize the structure of **1**. Then soft gel was prepared by adding **1** into cyclohexane directly. It shows remarkable thermoreversibility and can be demonstrated for several cycles. As is revealed by scanning electron microscopy (SEM) images, xerogel showed highly interconnected and homogeneous porous network structures, and hence, the gel is suitable for storage and controlled release.

## Introduction

The designed and prepared macrocyclic hosts [mainly including crown ethers (Liu et al., 2017; Morrison et al., 2017), cyclodextrins (Zhang et al., 2018; Larsen and Beeren, 2019), calixarenes (Wang et al., 2015; Tian et al., 2019), cucurbiturils (Kim et al., 2007; Wu et al., 2018; Xiao B. et al., 2019), and pillar[*n*]arenes (Xue et al., 2012; Sun et al., 2018; Chen et al., 2019; Ogoshi et al., 2019; Xiao T. et al., 2019)] and the investigations of their host–guest properties are the foundation of the development of supramolecular chemistry (Zheng et al., 2012; Yao et al., 2018; Zhang et al., 2019). As the third generation of macrocyclic compounds in supramolecular chemistry, calixarenes process several advantages, such as excellent flexibility, improved conformational mobility, and easy modification (Kim et al., 2012; Nimse and Kim, 2013).

*P*-tert-butyldihomooxacalix[4]arene is a well-known *p*-tert-butylcalix[4]arene analog in which one CH_2_ bridge is replaced by one -CH_2_OCH_2_- group (Marcos et al., 2002). Thus, dihomooxacalix[4]arene has a slightly larger cavity than that of calix[4]arene and usually possesses a more flexible cone conformation. What's more, the bridged oxygen atom might provide additional binding sites (Teixeira et al., 2017; An et al., 2018; Zhao et al., 2019). On the other hand, gels are interesting soft materials owing to their functional properties, leading to potential applications (Yao et al., 2017). Using gels as drug carriers has attracted tremendous attention for their numerous advantages in medical treatments, including prolonged drug release time, reduced side effects of drugs, and maintained effective plasma concentration (Nishimura et al., 2019; Teng et al., 2019; Thamizhanban et al., 2019). For example, Prof. Yao and co-workers prepared a soft gel based on pillar[5]arene by using a carbazone reaction and found that dyes such as TPP or TPPE can be incorporated into this gel and then released in a sustained way in water due to solvent exchange (Yao et al., 2017). However, investigations about supramolecular gels based on *p*-tert-butyldihomooxacalix[4]arene and their applications are rarely reported.

**Scheme 1:**
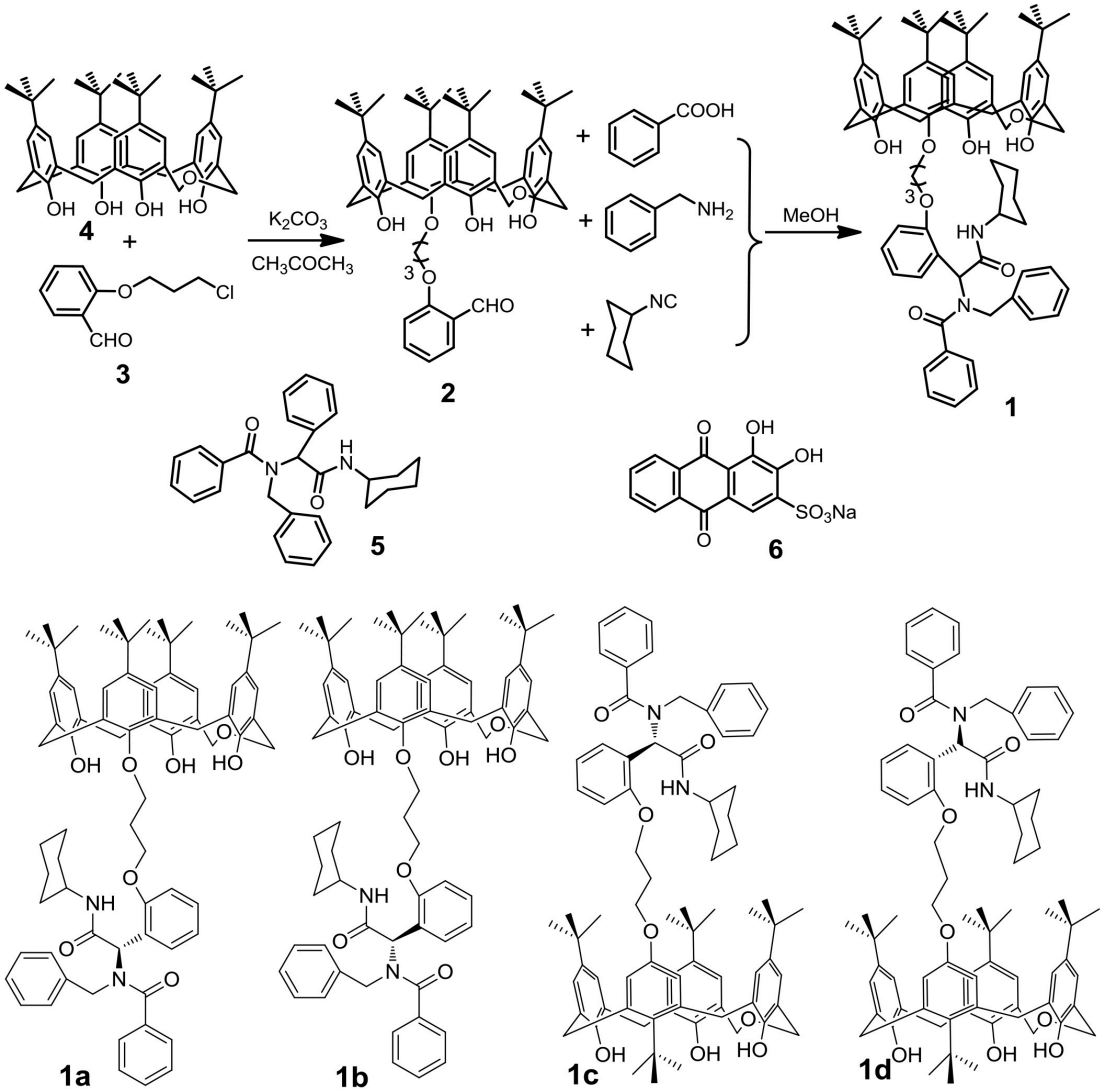
Synthetic route to *p*-tert-butyldihomooxacalix[4]arene **1** and chemical structures of compounds **5**, **6**, and **1**-isomers.

Herein, we designed and synthesized a novel functionalized *p*-tert-butyldihomooxacalix[4]arene **1** with two H-bonding sites through Ugi reaction (Scheme 1), which was prepared with good yield (70%). Then the soft gel was constructed by adding **1** into cyclohexane, heating the mixture, and leaving it cooled in the refrigerator for 2 min. **1-**based gel showed remarkable thermoreversibility, and this can be demonstrated for several cycles. The morphology of xerogel was revealed by scanning electron microscopy (SEM) images, which showed highly interconnected and homogeneous porous network structures. What's more, this gel can persist in its shape in water. Organic dyes such as alizarin red S **6** can be incorporated into this gel and are observed to be released in a sustained way in water. This may be very useful for preparing future smart materials by the implementation of related macrocyclic derivatives.

## Materials and Methods

### Synthesis of *p*-tert-Butyldihomooxacalix[4]-Arene 1

*P*-tert-butyldihomooxacalix[4]arene **4** (4.0 g, 5.9 mmol), Cl-alkoxy–substituted salicylaldehyde (1.8 g, 9.0 mmol), K_2_CO_3_ (1.2 g, 9.0 mmol), and KI (1.5 g) was added in 150 ml acetone. The mixture was stirred at 75°C for 24 h (Scheme S1). After removal of the inorganic salt, the solvent was evaporated, and the residue was purified by chromatography on silica gel (petroleum ether/ethyl acetate, *v*/*v* 5:1) to give **2** as a white solid (Liu et al., 2018). Then **2** (0.1 mmol, 0.885 g), benzyl amine (0.1 mmol, 0.107 g), benzoic acid (0.1 mmol, 0.122 g), and isocyancyclohexane (0.1 mmol, 0.109 g) were added into 7 ml methanol for reacting for 36 h. Then the solvent was evaporated, and the residue was purified by chromatography on silica gel (petroleum ether/ethyl acetate, v/v 3:1) to give **1** as a light yellow solid.

**1**: Yellow solid, 70%, m.p. 163.6–164.8°C; proton nuclear magnetic resonance spectroscopy (^1^H NMR) (400 MHz, CDCl_3_) (Figure S1) δ: *A-*isomer: 9.18 (brs, 1H, OH), 8.52 (s, 1H, OH), 7.74 (s, 1H, OH), 7.45–7.28 (m, 7H, phH), 7.26–7.82 (m, 15H, phH), 6.12 (s, 1H, CH), 5.23 (s, 1H, CH), 5.01–4.91 (m, 1H, CH_2_), 4.77–4.65 (m, 2H, CH_2_), 4.55–4.03 (m, 10H, CH_2_), 3.83–3.73 (m, 1H, CH_2_), 3.32–3.26 (m, 1H, CH_2_), 2.48–2.41 (m, 3H, CH_2_), 1.88–1.52 (m, 7H, CH_2_), 1.37–0.88 (m, 36H, CH_3_); *B-*isomer: 8.85 (s, 1H, OH), 6.28 (s, 1H, CH), 5.59 (s, 1H, CH); *C-*isomer: 8.33 (s, 1H, OH), 6.12 (s, 1H, CH), 5.48 (s, 1H, CH); *D-*isomer: 8.15 (s, 1H, OH), 5.69 (s, 1H, CH), 5.29 (s, 1H, CH); ratio of *A/B/C/D*-isomer = 0.2:0.1:0.1:0.1; ^1^H NMR (400 MHz, cyclohexane-d) δ: *A-*isomer: 9.50 (s, 1H, OH), 8.70 (s, 1H, OH), 7.90 (s, 1H, OH), 7.54–7.01 (m, 13H, phH), 6.87–6.72 (m, 9H, phH), 5.87–5.42 (m, 2H, CH), 4.88–3.91 (m, 14H, CH_2_), 3.67–3.08 (m, 6H, CH_2_), 2.48–2.37 (m, 2H, CH_2_), 1.86–1.45 (m, 6H, CH_2_), 1.23–1.18 (m, 27H, CH_3_), 1.17–1.15 (m, 9H, CH_3_); *B-*isomer: 8.33 (s, 1H, OH); ratio of *A/B*-isomer = 0.9:1 (Figure S9, Scheme S3); ^13^C NMR (100 MHz, CDCl_3_) (Figure S2) δ: 173.2, 157.4, 157.2, 152.7, 151.2, 149.1, 148.0, 147.8, 143.8, 142.7, 141.8, 141.7, 136.9, 132.8, 132.6, 131.9, 131.5, 130.3, 129.6, 128.4, 128.2, 128.2, 128.0, 127.8, 127.5, 127.5, 126.9, 126.9, 126.8, 126.8, 126.6, 126.0, 125.9, 125.8, 125.7, 125.3, 123.9, 122.8, 122.7, 120.7, 111.7, 77.4, 77.2, 77.0, 73.0, 72.6, 72.1, 71.7, 64.8, 64.4, 48.6, 34.3, 34.0, 33.9, 32.9, 32.8, 32.6, 31.7, 31.6, 31.5, 31.5, 31.5, 31.3, 30.3, 30.3, 30.1, 29.9, 29.8, 25.5, 25.1, 25.0; IR (KBr) υ: 3,386, 3,056, 2,959, 2,864, 1,735, 1,681, 1,637, 1,489, 1,453, 1,399, 1,361, 1,297, 1,246, 1,203, 1,077, 1,051, 876, 788, 735, 698, 596 cm^−1^; MS (*m/z*): HRMS (ESI) calcd. for C_76_H_92_N_2_O_8_Na ([M+Na]^+^): 1183.6751, found: 1183.6797 (Figure S3).

### Materials

All reagents and solvents were commercially available in analytical grade and used as received. Further purification and drying by standard methods were employed, and distillation was done prior to use when necessary. All evaporations of organic solvents were carried out with a rotary evaporator in conjunction with a water aspirator. *p*-tert-Butyldihomooxacalix[4]arenes were prepared according to published methods (Marcos et al., 2002). Melting point measurements were taken on a hot-plate microscope apparatus and are uncorrected. ^1^H and ^13^C NMR spectra were recorded with an Aviance III 400 MHz or 600 MHz liquid-state NMR spectrometer. IR spectra were obtained on a Bruker Tensor 27 spectrophotometer (KBr disk). HRMS was determined on a Bruker maXis mass spectrometer. Fluorescence spectra were recorded on a Shimadzu HITACHI F-4500 spectrophotometer. Rheological studies were performed on an AR-G2 rheometer (TA Instruments, USA) using a plate–plate geometry. The SEM image was obtained from a ZEISS Gemini SEM 300 instrument.

## Results and Disscussion

### Gelation Tests

**Figure 1:**
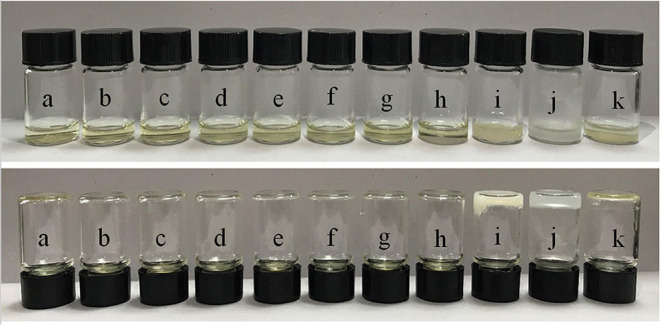
Gelation test of compound **1** in different solvents: **(a)** methanol; **(b)** ethanol; **(c)** pentanol; **(d)** tert-butanol; **(e)** acetonitrile; **(f)** ethyl acetate; **(g)** tetrahydrofuran; **(h)** toluene; **(i)** cyclopentane; **(j)** cyclohexane; and **(k)** hexane.

The gelation test results obtained for calix[4]arene **1** in different solvents are shown in Figure 1. We chose methanol, ethanol, pentanol, *tert*-butanol, acetonitrile, ethyl acetate, tetrahydrofuran, toluene, cyclopentane, cyclohexane, and hexane as the solvents and found that **1** can disperse well in all these solvents with the concentration at about 100 mmol at 40°C. However, when the temperature cooled to 25°C, **1** could form a gel in cyclohexane (Figure 1, under, sample j) but could not form a gel in methanol, ethanol, pentanol, tert-butanol, acetonitrile, ethyl acetate, tetrahydrofuran, toluene, and hexane, as the samples flowed under gravity (Figure 1, under, samples a, b, c, d, e, f, g, h, k). Sample i seemed to be gelled but also flowed under slight vibrations. For comparison, compound **5** in Scheme S2 (Figures S4 and S5) without the calix[4]arene framework could not form a gel in the same condition, indicating that the calix[4]arene framework is an integral part of the gel formation process. Further investigation found that the critical concentration of compound **1** to form a gel in cyclohexane is 10.9 wt%. It should be pointed out that the compound **1** we used to construct gel contains both conformers.

### ^1^H NMR Studies

**Figure 2:**
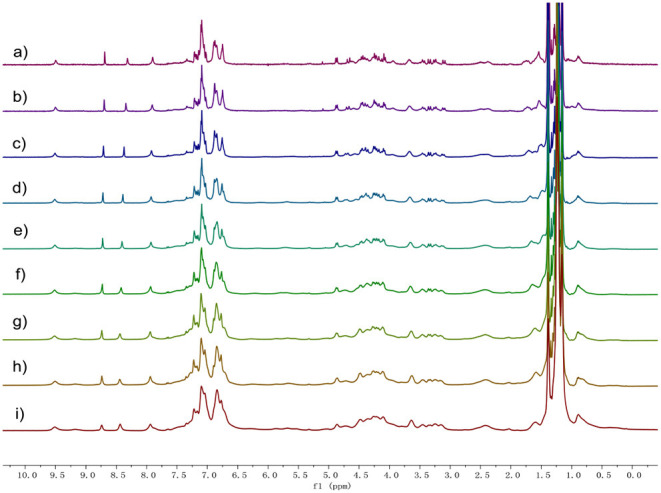
Proton nuclear magnetic resonance spectroscopy (^1^H) NMR spectra (400 MHz, cyclohexane-d, 20°C) of **1** at different concentrations: **(a)** 5, **(b)** 10, **(c)** 20, **(d)** 30, **(e)** 40, **(f)** 50, **(g)** 60, **(h)** 70, and **(i)** 80 mM.

In order to investigate the intermolecular interactions during gel formation, ^1^H NMR and 2D diffusion-ordered ^1^H NMR spectroscopy (DOSY) were performed. As shown in Figure 2, ^1^H NMR spectra of **1** in *d*-cyclohexanes were recorded over the concentration range of 5.00 up to 80 mM. As the concentration increased, all the signals of protons on compound **1** became broad, which demonstrated the formation of high-molecular-weight aggregates (Yan et al., 2012).

DOSY showed that the weight-average diffusion coefficient (*D*) of **1** in cyclohexane-d decreased gradually from 2.03 × 10^−10^ to 3.15 × 10^−11^ m^2^ s^−1^ upon the concentration of **1** increasing from 5.0 up to 80 mM (Figure S7, ESI†). FT-IR investigation confirmed the formation of H-bond after **1** self-assembly into gel (Figure S8). These observations proved that there is an increase in the average aggregation size owing to the concentration going on, indicating the formation of polymeric structures in cyclohexane.

### Rheological Properties

**Figure 3:**
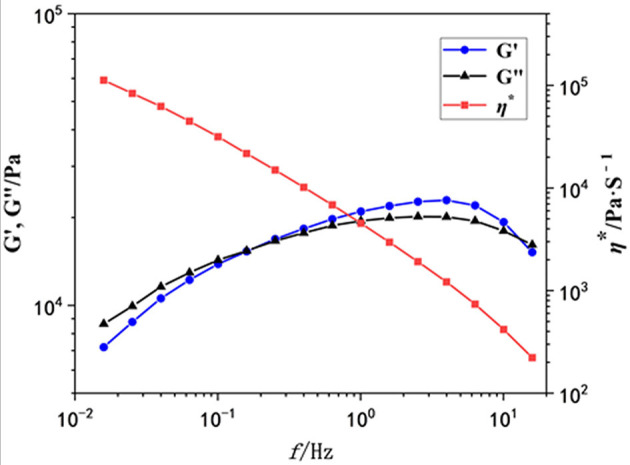
The rheological property of the gel as a function of the scanning frequency (Hz). G′: blue points; G″, black points; η*, red points.

Then we used oscillatory rheological characterization to investigate the mechanical properties of this gel in detail. The storage (G′) and loss (G″) moduli of the obtained gel as a function of the scanning frequency (*f*) were investigated. As shown in Figure 3, when the *f* increased to 0.2, the intersection point of G′ and G″ (G′ = G″) appeared, indicating the formation of a gel. However, G′ is larger than G″ at frequencies from 0.2 to 20 Hz, and both G′ and G″ are independent of the frequency, indicating the existence of network structures in the gel. The value of G′ is about 10,000 Pa, so this gel exhibits moderate mechanical properties. Additionally, the viscometry (η^*^, red line) decreased sharply with the scanning frequency increasing.

### SEM

**Figure 4:**
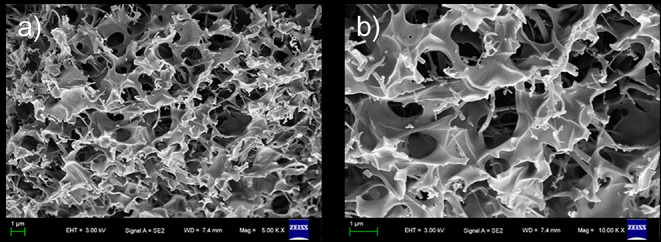
Scanning electron microscopy (SEM) images of three-dimensional network of **1**-based xerogel. **(b)** is partial enlarge of **(a)**.

The morphology of this xerogel, which was obtained using a freeze-drying methodology to remove cyclohexane, was then examined by SEM. As shown in Figure 4, SEM revealed that the xerogel was an interconnected honeycomb-like porous structure in which large open plate-like structures and fibrils with diameters of 200 nm and lengths of several micrometers aggregated into very distinct micro-structured networks. It is worth pointing out that porous materials have attracted a great deal of interest in both science and technology due to their potential applications in many areas. Interestingly, at room temperature, our gel was stable for about 2 months in aqueous solution (pH from 3 to 11, Figure S6). Furthermore, the fresh gel can persist in its shape after pressing by heavy weight.

### Sustained Release

**Figure 5:**
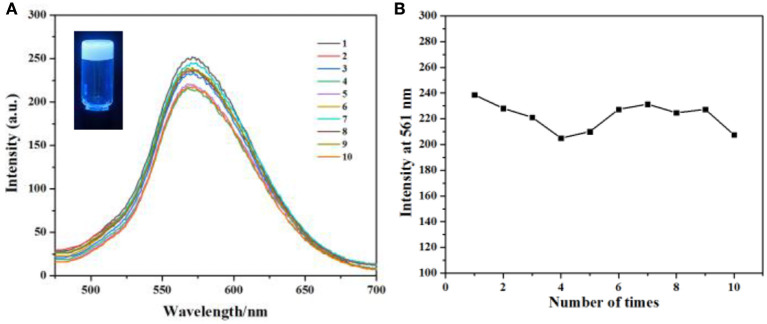
**(A)** Fluorescence emission spectra (the excitation wavelength is 365 nm) of **6** loaded gel vs. number of times water was changed. **(B)** The maximum intensity of **(A)** with the extraction times.

As we all known, gel is a new type of soft material, has obtained great interest from both chemistry and materials scientists, and has shown useful applications in various areas. When gel is applied in drug release, the major disadvantage is that most drugs will release in a rapid and complete way. In this condition, the concentration of the drug could not maintain a good value, so the efficiency of the uptake of the drug is very low. However, our supramolecular gel can incorporate some small molecules and then release them in a sustained way in water. So our gel can be used in sustained drug release for cancer therapy with good efficiency. Herein, alizarin red S **6** was used as a model compound to investigate the potential of our gel as a platform for sustained drug release. **6** can be incorporated in our soft gel to form a fluorescence gel (Figure 5, inset). Then when we immersed this gel in water and changed the water every 12 h, the fluorescence intensity of the solution also remained a certain value after repeating 10 times (Figure 5), indicating that **6** was released from calixarene-based gel in a sustained way.

## Conclusions

In this paper, we synthesized a new calix[4]arene **1** through Ugi reaction, which was prepared with good yield (70%), starting from condensed *p*-tert-butyldihomooxacalix[4]arene O-alkoxy–substituted benzaldehydes, benzoic acid, benzylamine, and cyclohexyl isocyanide. Then soft gel was prepared by adding the **1** into cyclohexane directly through a heating/cooling process. The gel shows remarkable thermoreversibility, and this can be demonstrated for several cycles. ^1^H NMR, FT-IR, DOSY, rheological characterization, FL, and SEM were employed to study the formation process and resultant gel. Furthermore, compound **6** as a model drug can be incorporated into our supramolecular gel and was observed to be released in a sustained way in water. This may have potential applications in sustained drug release for cancer therapy. Our next study will focus on cell and animal experiments of this gel in sustained drug release for cancer therapy.

## Data Availability Statement

The raw data supporting the conclusions of this article will be made available by the authors, without undue reservation, to any qualified researcher.

## Author Contributions

Individual authors contributed to the present paper as follows: HG and RZ prepared all the compounds. YH and CY analyzed the data. JW and CY wrote the paper.

### Conflict of Interest

The authors declare that the research was conducted in the absence of any commercial or financial relationships that could be construed as a potential conflict of interest.

## References

[B1] An L. Wang J.-W. Wang C. Zhou S.-S. Sun J. Yan C.-G. (2018). 2, 3-Ethylene-bridged dihomooxacalix[4]arenes: synthesis, X-ray crystal structures and highly selective binding properties with anions. New J. Chem. 42:10689 10.1039/C8NJ01284A

[B2] Chen J. Wang Y. Wang C. Long R. Chen T. Yao Y. (2019). Functionalization of inorganic nanomaterials with pillar[*n*]arenes. Chem. Commun. 55, 6817–6826. 10.1039/c9cc03165k 31139803

[B3] Kim H. J. Lee M. H. Mutihac L. Vicens J. Kim J. S. (2012). Host-guest sensing by calixarenes on the surfaces. Chem. Soc. Rev. 41, 1173–1190. 10.1039/c1cs15169j 21870018

[B4] Kim K. Selvapalam N. Ko Y. H. Park K. M. Kim D. Kim J. (2007). Functionalized cucurbiturils and their applications. Chem. Soc. Rev. 36, 267–279. 10.1039/b603088m 17264929

[B5] Larsen D. Beeren S. R. (2019). Enzyme-mediated dynamic combinatorial chemistry allows out-of-equilibrium template-directed synthesis of macrocyclic oligosaccharides. Chem. Sci. 10, 9981–9987. 10.1039/C9SC03983J 32055354 PMC6979337

[B6] Liu Z. Nalluri S. K. M. Stoddart J. F. (2017). Surveying macrocyclic chemistry: from flexible crown ethers to rigid cyclophanes. Chem. Soc. Rev. 46, 2459–2478. 10.1039/c7cs00185a 28462968

[B29] Liu Y. Zhao L.-L. Sun J. Yan C.-G. (2018). Convenient synthesis and coordination properties of *p*-*tert*-Butyldihomooxacalix[4]arene mono-schiff bases. Polycyclic Aromat. Compd. 40, 644–659. 10.1080/10406638.2018.1469520

[B7] Marcos P. Ascenso J. Pereira J. L. C. (2002). Synthesis and NMR conformational studies of p-tert-butyldihomooxacalix[4]arene derivatives bearing pyridyl pendant groups at the lower rim. Eur. J. Org. Chem. 2002, 3034–3041. 10.1002/1099-0690(200209)2002:17<3034::AID-EJOC3034>3.0.CO;2-I

[B8] Morrison P. W. J. Porfiryeva N. N. Chahal S. Salakhov I. A. Lacourt C. Semina I. I.. (2017). Crown ethers: novel permeability enhancers for ocular drug delivery? Mol. Pharmaceutics 14, 3528–3538. 10.1021/acs.molpharmaceut.7b00556 28825493

[B9] Nimse S. B. Kim T. (2013). Biological application of functionalized calixarenes. Chem. Soc. Rev. 42, 366–386. 10.1039/C2CS35233H 23032718

[B10] Nishimura T. Sumi N. Mukai S.-A. Sasaki Y. Akiyoshi K. (2019). Supramacromolecular injectable hydrogels by crystallization-driven self-assembly of carbohydrate-conjugated poly(2-isopropyloxazoline)s for biomedical applications. J. Mater. Chem. B 7, 6362–6369. 10.1039/c9tb00918c 31642846

[B11] Ogoshi T. Kakuta T. Yamagishi T. A. (2019). Application pf pillar[n]arene-based supramolecular assemblies. Angew. Chem. Ind. Ed. 58, 2197–2206. 10.1002/anie.201805884 29900642

[B12] Sun S. Geng M. Huang L. Chen Y. Cen M. Lu D.. (2018). A new amphiphilic pillar[5]arene: synthesis and controllable self-assembly in water and application in white-light-emitting systems. Chem. Commun. 54, 13006–13009. 10.1039/c8cc07658h 30393794

[B13] Teixeira F. A. Marcos P. M. Ascenso J. R. Brancatelli G. Hickey N. Geremia S. (2017). Selective binding of spherical and linear anions by tetraphenyl(thio)urea-based dihomooxacalix[4]arene receptors. J. Org. Chem. 21, 11383–11390. 10.1021/acs.joc.7b01801 28990384

[B14] Teng L. Chen Y. Jia Y.-G. Ren L. (2019). Supramolecular and dynamic covalent hydrogel scaffolds: from gelation chemistry to enhanced cell retention and cartilage regeneration. J. Mater. Chem. B 7, 6705-−6736. 10.1039/c9tb01698h 31647089

[B15] Thamizhanban A. Lalitha K. Sarvepalli G. P. Maheswari C. U. Sridharan V. Rayappan J. B. B.. (2019). Smart supramolecular gels of enolizable amphiphilic glycosylfuran. J. Mater. Chem. B. 7, 6238–6246. 10.1039/c9tb01480b 31566636

[B16] Tian H.-W. Liu Y.-C. Guo D.-S. (2019). Assembling features of calixarene-based amphiphilie and supra-amphiphiles. Mater. Chem. Front. 4, 46–98. 10.1039/C9QM00489K

[B17] Wang Y.-X. Zhang Y.-M. Liu Y. (2015). Photolysis of an amphiphilic assembly by calixarene-induced aggregation. J. Am. Chem. Soc. 137, 4543–4549. 10.1021/jacs.5b01566 25803552

[B18] Wu W. Song S. Cui X. Sun T. Zhang J.-X. Ni X.-L. (2018). pH-Switched fluorescent pseudorotaxane assembly of cucurbit[7]uril with bispyridinium ethylene derivatives. Chin. Chem. Lett. 29, 95–98. 10.1016/j.cclet.2017.08.049

[B19] Xiao B. Wang Q. Zhang S. Li X.-Y. Long S.-Q. Xiao Y.. (2019). Cucurbit[7]uril-anchored polymer vesicles enhance photosensitization in the nucleus. J. Mater. Chem. B 7, 5966–5971. 10.1039/c9tb01526d 31524915

[B20] Xiao T. Qi L. Zhong W. Lin C. Wang R. Wang L. (2019). Stimuli-responsive nanocarriers constructed from pillar[*n*]arene-based supra-amphiphiles. Mater. Chem. Front. 3, 1973–1993. 10.1039/C9QM00428A

[B21] Xue M. Yang Y. Chi X. Zhang Z. Huang F. (2012). Pillararenes, a new class of macrocycles for supramolecular chemistry. Acc. Chem. Res. 45, 1294–1308. 10.1021/ar2003418 22551015

[B22] Yan X. Xu D. Chi X. Chen J. Dong S. Ding X.. (2012). A multiresponsive, shape-persistent, and elastic supramolecular polymer network gel constructed by orthogonal self-assembly. Adv. Mater. 24, 362–369. 10.1002/adma.201103220 22161963

[B23] Yao Y. Sun Y. Yu H. Chen W. Dai H. Shi Y. (2017). A pillar[5]arene based gel from a low molecular weight gelator for sustained dye release in water. Dalton Trans. 46, 16802–16806. 10.1039/c7dt04001f 29171609

[B24] Yao Y. Zhao R. Shi Y. Cai Y. Chen J. Sun S.. (2018). 2D amphiphilic organoplatinum(ii) metallacycles: their syntheses, self-assembly in water and potential application in photodynamic therapy. Chem. Commun. 54, 8068–8071. 10.1039/c8cc04423f 29968880

[B25] Zhang R. Wang C. Long R. Chen T. Yan C. Yao Y. (2019). Pillar[5]arene based [1]rotaxane systems with redox-responsive host-guest property: design, synthesis and the key Role of chain length. Front. Chem. 7:508. 10.3389/fchem.2019.00508 31396505 PMC6663970

[B26] Zhang Y.-M. Zhang N.-Y. Xiao K. Yu Q.-L. Liu Y. (2018). Photo-controlled reversible microtubule assembly mediated by paclitaxel-modified cyclodextrin. Angew. Chem. Int. Ed. 57, 8649–8653. 10.1002/anie.201804620 29781242

[B27] Zhao L.-L. Yang X.-S. Chong H. Wang Y. Yan C.-G. (2019). Multi-point interaction-based recognition of fluoride ions by tert-butyldihomooxacalix[4]arenes bearing phenolic hydroxyls and thiourea. New J. Chem. 43, 5503–5511. 10.1039/C8NJ06333H

[B28] Zheng B. Wang F. Dong S. Huang F. (2012). Supramolecular polymers constructed by crown ether-based molecular recognition. Chem. Soc. Rev. 41, 1621–1636. 10.1039/c1cs15220c 22012256

