# Corrigendum: A *p-tert*-Butyldihomooxacalix[4]arene Based Soft Gel for Sustained Drug Release in Water

**DOI:** 10.3389/fchem.2020.00721

**Published:** 2020-08-21

**Authors:** Hao Guo, Runmiao Zhang, Ying Han, Jin Wang, Chaoguo Yan

**Affiliations:** ^1^School of Chemistry and Chemical Engineer, Yangzhou University, Yangzhou, China; ^2^School of Chemistry and Chemical Engineer, Nantong University, Nantong, China

**Keywords:** calixarenes, gel, controlled release, macrocyclic compounds, Ugi reaction

In the original article, there was an error. In the title, “*p*-tert-Tutyldihomooxa-calix[4]arene” was misspelled. A correction has been made to the title which should read “A *p-tert*-Butyldihomooxacalix[4]arene Based Soft Gel for Sustained Drug Release in Water”.

There was an error in the abstract: “one CH_2_ bridge is replaced by one -O- group” was misspelled. A correction has been made to the first sentence abstract which should read as follows: “*P*-*tert*-butyldihomooxacalix[4]arene is a well-known calix[4]arene analog in which one CH_2_ bridge is replaced by one -CH_2_OCH_2_- group.”

Also, “one CH_2_ bridge is replaced by one -O- group” was misspelled in Introduction, in the first sentence of the second paragraph. The corrected sentence should read as follows: “*P*-tert-butyldihomooxacalix[4]arene is a well-known *p*-tert-butylcalix[4]arene analog in which one CH_2_ bridge is replaced by one -CH_2_OCH_2_- group (Marcos et al., 2002).”

In the original article, the following reference was not cited in the article: Liu, Y., Zhao, L.-L., Sun, J., and Yan, C.-G. (2018). Convenient synthesis and coordination properties of *p*-*tert*-butyldihomooxacalix[4]arene mono-schiff bases. *Polycyclic Aromat. Compd*. 40, 644–659. doi: 10.1080/10406638.2018.1469520. The citation has now been inserted in Materials and Methods, in section Synthesis of p-tert-Butyldihomooxacalix[4]-Arene 1, in the first paragraph which should read:

*P*-tert-butyldihomooxacalix[4]arene **4** (4.0 g, 5.9 mmol), Cl-alkoxy–substituted salicylaldehyde (1.8 g, 9.0 mmol), K_2_CO_3_ (1.2 g, 9.0 mmol), and KI (1.5 g) was added in 150 ml acetone. The mixture was stirred at 75°C for 24 h (Scheme S1). After removal of the inorganic salt, the solvent was evaporated, and the residue was purified by chromatography on silica gel (petroleum ether/ethyl acetate, *v*/*v* 5:1) to give **2** as a white solid (Liu et al., 2018). Then **2** (0.1 mmol, 0.885 g), benzyl amine (0.1 mmol, 0.107 g), benzoic acid (0.1 mmol, 0.122 g), and isocyancyclohexane (0.1 mmol, 0.109 g) were added into 7 ml methanol for reacting for 36 h. Then the solvent was evaporated, and the residue was purified by chromatography on silica gel (petroleum ether/ethyl acetate, v/v 3:1) to give **1** as a light yellow solid.

Additionally, in the original article, Scheme 1 indicates compound **1** in the cone and partial cone conformations, but these two conformers are not explained. A correction has been made to Results and Discussion, section Gelation tests, end of the second paragraph by adding the following sentence: “It should be pointed out that the compound **1** we used to construct gel contains both conformers.”

The authors apologize for these errors and state that these do not change the scientific conclusions of the article in any way. The original article has been updated.
