# Corrigendum: A *p-tert*-Butyldihomooxacalix[4]arene Based Soft Gel for Sustained Drug Release in Water

**DOI:** 10.3389/fchem.2020.624978

**Published:** 2020-12-16

**Authors:** Hao Guo, Runmiao Zhang, Ying Han, Jin Wang, Chaoguo Yan

**Affiliations:** ^1^School of Chemistry and Chemical Engineer, Yangzhou University, Yangzhou, China; ^2^School of Chemistry and Chemical Engineer, Nantong University, Nantong, China

**Keywords:** calixarenes, gel, controlled release, macrocyclic compounds, Ugi reaction

In the original Supporting Information, we missed Scheme S2, Scheme S3, and Figures S6, S7, S8, S9, please update it with the new ESI document we provided below.

The authors apologize for this error and state that this does not change the scientific conclusions of the article in any way. The original article has been updated.

