# Supplementary material for: A p-tert-Butyldihomooxacalix[4]arene Based Soft Gel for Sustained Drug Release in Water
Source: Front Chem. 2020 Feb 28;8:33. doi: 10.3389/fchem.2020.00033 (PMC7059609; doi:10.3389/fchem.2020.00033)
Supplement: Supplementary file 1 [file Data_Sheet_1.docx]

A *p-tert-*Butyldihomooxacalix[4]arene based soft gel for sustained drug release in water

*Hao Guo1, 2, Runmiao Zhang1, 2, Ying Han1*, Jin Wang2*, and Chaoguo Yan1*

*1 School of Chemistry and Chemical Engineer, Yangzhou University, Yangzhou, Jiangsu, 225000, P.R. China,*

*2 School of Chemistry and Chemical Engineer, Nantong University, Nantong, Jiangsu, 226019, P.R. China,*

1. Synthesis of *p*-tert-butyldihomooxacalix[4]arene **1**

Scheme S1. Synthetic route to *p*-tert-butyldihomooxacalix[4]arene **1**.

***Figure S1*** ^1^H NMR spectrum (400 MHz, CDCl_3_, 293 K) of **1**.

***Figure S2*** ^13^C NMR spectrum (100 MHz, CDCl_3_, 293 K) of **1**.

***Figure S3*** Mass spectra of **1** C_76_H_92_N_2_O_8_Na ([M+Na]^+^): 1183.6751, found: 1183.6797.

1. Synthesis of compound **5**^S1^

Scheme S2. Synthetic route to compound **5**.

***Figure S4*** ^1^H NMR spectrum (400 MHz, CDCl_3_, 293 K) of **5**.

***Figure S5*** ^13^C NMR spectrum (100 MHz, CDCl_3_, 293 K) of **5**.


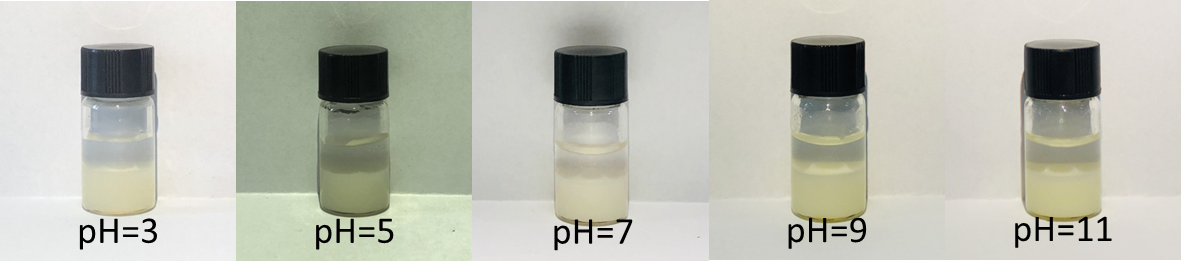


***Figure S6*** Stability of our supramolecular gel in aqueous solution with different pH value.


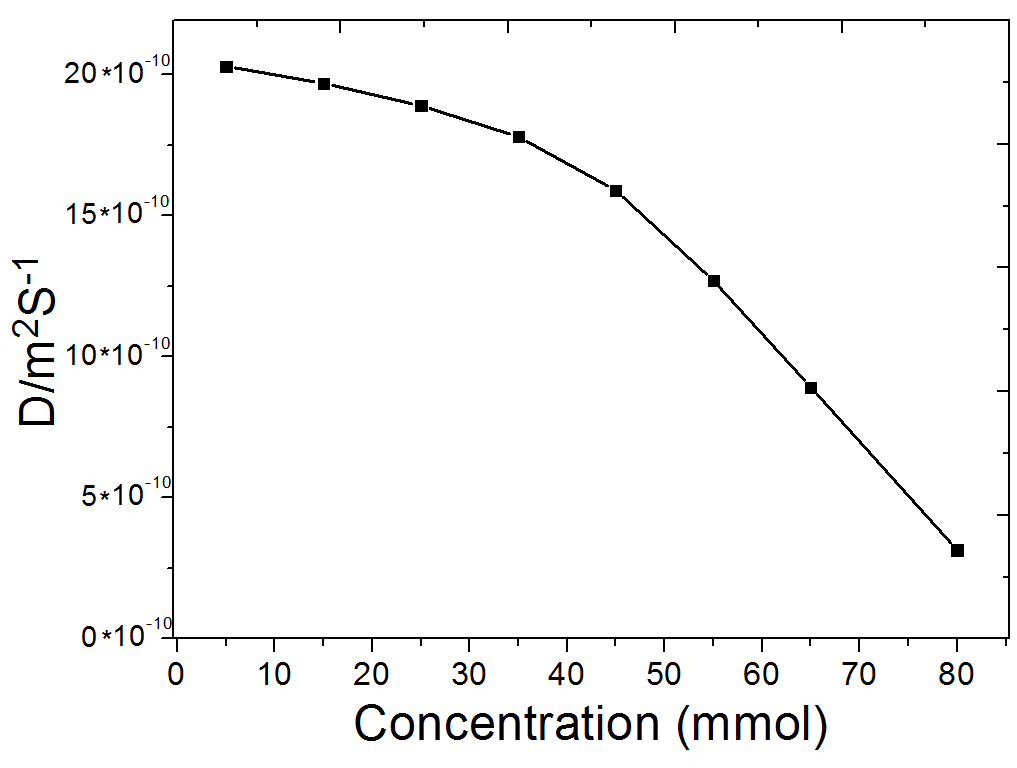


***Figure S7*** Concentration dependence diffusion coefficient D (500 MHz, d-cyclohexane, 293 K) of compound **1**.


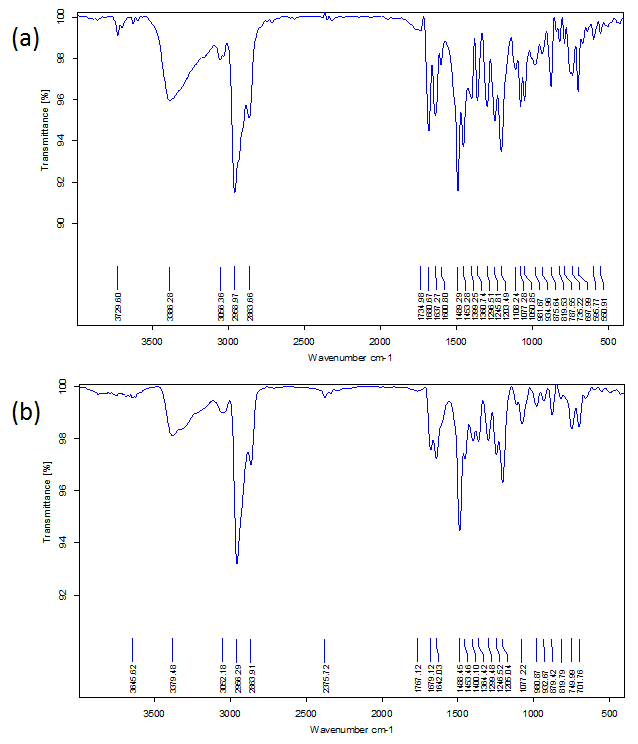


***Figure S8*** FT-IR spectra of (a) pure compound **1** and (b) **1**-based xerogel.

***Figure S9*** ^1^H NMR spectrum (400 MHz, d-cyclohexane, 293 K) of **1**.

***Scheme S3*** Chemical structures of two isomers of **1** in cyclohexane.

S1. C.-P. Dong, A. Uematsu, S. Kumazawa, Y. Yamamoto, S. Kodama, A. Nomoto, M. Ueshima, A. Ogawa, *J. Org. Chem.*, **2019**, *84*, 11562−11571.
